# A histological ontology of the human cardiovascular system

**DOI:** 10.1186/s13326-017-0158-5

**Published:** 2017-10-02

**Authors:** Claudia Mazo, Liliana Salazar, Oscar Corcho, Maria Trujillo, Enrique Alegre

**Affiliations:** 1 Computer and Systems Engineering School, Universidad del Valle, Cali, Colombia; 2 Morphology Department, Faculty of Health, Universidad del Valle, Cali, Colombia; 3 Ontology Engineering Group, Universidad Politécnica de Madrid, Madrid, Spain; 4 Industrial and Informatics Engineering School, Universidad de León, León, Spain

**Keywords:** Ontology, Human histology, Fundamental tissues, Organs, Cardiovascular system

## Abstract

**Background:**

In this paper, we describe a histological ontology of the human cardiovascular system developed in collaboration among histology experts and computer scientists.

**Results:**

The histological ontology is developed following an existing methodology using Conceptual Models (CMs) and validated using *OOPS!*, expert evaluation with CMs, and how accurately the ontology can answer the Competency Questions (CQ). It is publicly available at http://bioportal.bioontology.org/ontologies/HO and https://w3id.org/def/System.

**Conclusions:**

The histological ontology is developed to support complex tasks, such as supporting teaching activities, medical practices, and bio-medical research or having natural language interactions.

## Background

Morphological science’s experts knowledge is an important source in histology studies and practices for human studies at cellular, tissue, organ and system levels. As many other domains, histology domain also suffers from problems like vocabulary heterogeneity, the use of ambiguous language, semantic differences and subjectivity that may affect research, analysis and information retrieval processes. Different terms are used to designate the same concept –or structure– or the same term is used with different meanings, in different texts.

Two main challenges are identified in the histology domain [1]: (i) communicate specifically, clearly and precisely histology concepts and (ii) represent or model knowledge from histology data sources in order to interact and process it automatically. These challenges require a profound analysis of the structure and the concepts of histological terminologies. This analysis can be done by constructing histological domain ontologies. The use of ontologies for representing knowledge is common in medical applications, such as anatomy, and histology among others. The union between ontologies and medical information is considered as a necessary alternative to solve main problems regarding those sources of information [2–4].

The term “ontology” has many definitions depending on the author and the way an ontology is built and used by computer systems. One of the most widespread definition of ontology is: “Ontology is an explicit and formal specification of a shared conceptualisation” [5]. Ontologies create models to formalise knowledge in the same way that it is used. From a histology perspective, an ontology would consist of concepts defined by histological knowledge. Additionally, relations, attributes, rules and axioms enrich and contribute to expand the vocabulary used to formalise knowledge. On the other hand, a taxonomy is a set of definitions that are organised by a hierarchy that starts at the most general description and gets more refined and specific terms as the hierarchy goes down.

Many ontologies and taxonomies are available in electronic form with Open Source licenses. Ones of the best known medical taxonomies are: GALEN [6] (basic clinical concepts — fracture, bone, and so on — controlling combinations of related concepts — bone fractures — and complex concepts — clavicle fracture), UMLS (Unified Medical Language System) [7], MeSH (Medical Subject Heading) [8], Kingsbury Center for Cancer Care Glossary [9], MedicineNet Medical Dictionary [10], Multilingual Glossary of Technical, and Popular Medical Terms in nine European Languages [11], ICD (International Classification of Diseases) [12] among others [13]. Some ontologies are used in web retrieval systems [14], identification of relations between diseases [15], and diagnosis [16], among others [13]. Some ontologies are used in web retrieval systems [14], identification of relations between diseases [15], and diagnosis [16], among others. Uberon ontology is an anatomy ontology, which is a common standard used by the biomedical research community [17]. However, none of these ontologies covers histological knowledge of the human cardiovascular system without pathologies in the same kind of guidance and organisation to our research.

In this paper, we describe our work to build a histological ontology of the human cardiovascular system. This work is licensed under a Creative Commons Attribution-NonCommercial-ShareAlike 4.0 Generic^1^ license. We selected the cardiovascular system because it is one of the most committed to the development of diseases associated with modern life. To the best of our knowledge, and after a careful search in the most relevant repositories, there is no a histological ontology in the literature, thus we consider this one to be a relevant contribution to the research community in the histology domain. We left the histological ontology publicly available at http://bioportal.bioontology.org/ontologies/HO, the documentation at https://w3id.org/def/System and the OWL files at https://github.com/claxima/HistologicalOntology.

The rest of the paper is structured as follows: the methodology to build the histological ontology is presented in “Methods” section; the evaluation and the results are presented and discussed in “Results” section; in “Discussion” section we analyse the obtained results; and some conclusions are presented in “Conclusions” section.

## Methods

**Fig. 1:**
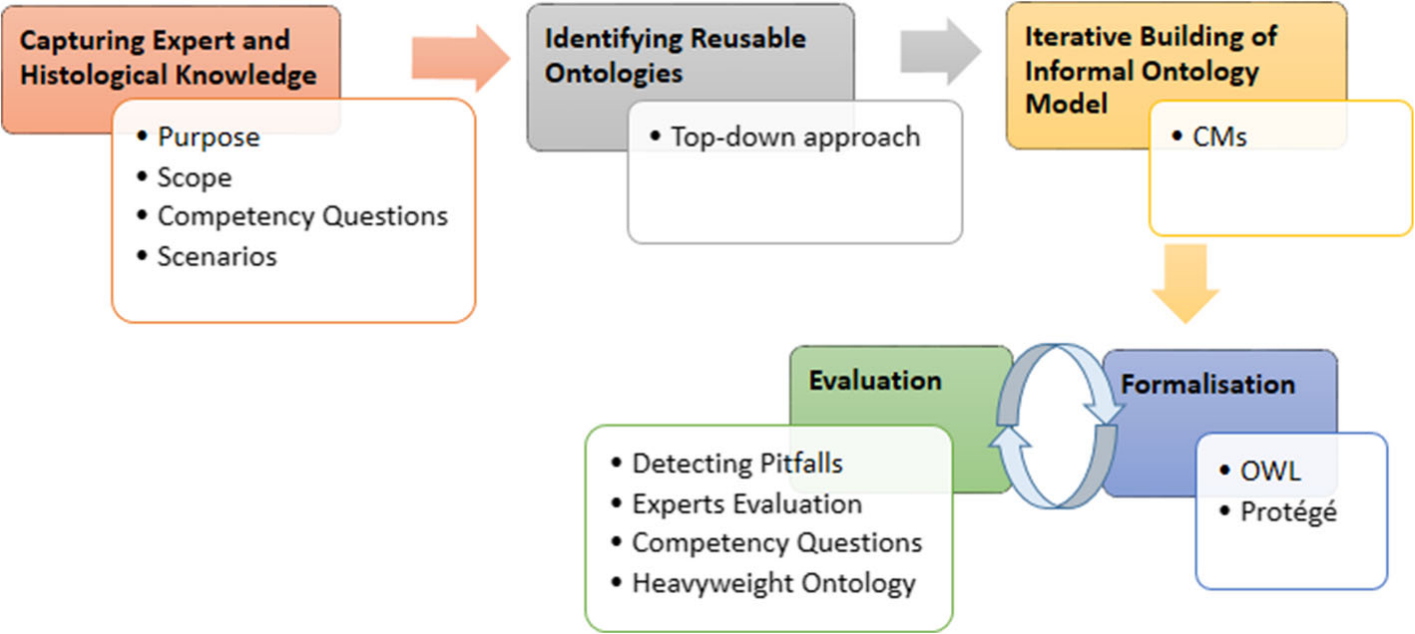
Methodology to develop ontologies

The NeOn methodology is one of the most used methodologies for ontology engineering [18]. This methodology does not prescribe a rigid ontology development workflow, but instead it suggests nine scenarios for developing ontologies. The methodology covers commonly occurring situations which mostly focus on reusing, merging, restructuring and re-engineering ontological resources. Taking into account that we will create a histological ontology without reusing ontological resources, according to our analysis of the State-of-the-Art, we decided to use the methodology proposed in [19]. This methodology consists of the following steps: (i) identification of purpose, scope, CQs and scenarios, (ii) identification of those ontologies we could reuse, (iii) domain analysis and knowledge acquisition, (iv) iterative building of informal ontology models, (v) formalisation and (vi) evaluation. We modify minimally this methodology in steps i, iii and vi, Fig. 1 presents the resulting steps. Firstly, we merge step (i) and (iii) which will be our first step called capturing expert and histological knowledge. Secondly, we use three evaluation criteria — detecting pitfalls, expert evaluation and answering CQs — while [19] uses two evaluation criteria — CMs and the Protégé axiom language plug-in provided by Protégé.

### Capturing expert and histological knowledge

In this step, the aim is domain knowledge extraction using a set of knowledge capture activities — meetings, discussions, histology classes, among others. We planned a series of activities with the experts through which the foundations of our ontology were built: purpose, scope, CQs and scenarios. We hosted a series of meetings with the group of histology experts conformed by members of the research group Teblami^2^, from the Universidad del Valle^3^, in which the domain experts discussed the terminology and the structure used to describe the processes to analyse a histological sample. The experts team was comprised by histology professors, with more than five years of teaching and research experience, and biomedical graduate students, with mayor on histology, all of them formed the research histology area.

The questions to answer, at this stage, were the following: (i) what is the ontology going to be used for?, (ii) what do we want the ontology to be aware of?, (iii) what is the scope of the knowledge that we want to have in the ontology?, and (iv) how is the ontology going to be used?, the answers are provided in the following subsections.

#### Purpose, Scope and Scenarios

Commonly, ontology development is not the final goal of the process. Instead, an ontology becomes an artefact to be used by other systems. Under this perspective, the purpose is defined by the main reasons that can lead to creating an ontology [20]. Our Ontology was constructed for: (i) sharing a common understanding of histology knowledge between people and machines in processes such as automatic recognition and identification of cells, tissues and organs; (ii) allowing reuse of domain knowledge; (iii) allowing change specifications of histology knowledge, if changes occur in it. Therefore, our main target community are both, medical professors and biomedical researchers. In addition, explicit specifications of histology knowledge are useful for users who should learn the meaning of histological terms, to specialised users that want to develop a semantic visual information retrieval system, or to other users that want to label automatically histological images or teach to students histological’s structures and relations.

This work is focused on the human cardiovascular system, which is one of the most committed to the development of diseases associated with modern life. Three scenarios are described to illustrate and motivate the use for this histological ontology. These scenarios are later used to develop a set of CQs and to indicate how the ontology would be used in these cases.

##### Professor:

a histology expert works as a professor in a university teaching histology of the cardiovascular system. The expert teaches different group levels, covering histology of cells, tissues, organs and systems. The professor should cover each topic considering components, relations and organisations. Additionally, she/he may also promote self-learning to on-campus students and facilitate on-line learning to external or remote students.

##### Biomedical research:

a researcher is interested in working with a big data set of histological images, which are not labelled. The researcher has to label each histological image with cells, tissue and organs using a controlled vocabulary, in short time, reducing subjectivity and increasing precision. Additionally, the researcher should search and recover images according to present structures to develop different steps in her or his research.

##### Medical:

a histology expert works in a hospital analysing samples in the cardiovascular system context. When receiving a sample, the histologist analyses, labels and validates different characteristics of the sample.

Having defined the purpose, scope, and scenarios of the ontology, we discussed the CQs with our histology experts. These CQs were used at a later stage in order to evaluate the resulting ontology.

#### Competency questions (CQs)

CQs are the kind of questions for which we want the ontology to be able to provide support for representation or reasoning processes. Additionally, those questions are essential for evaluating ontologies [21]. Experts should express the CQs in natural language without any constraint. Based on the above scenarios, we have identified four categories of CQs: classifications, properties, constraints and inferences. Examples of those CQs are presented in Table 1, and https://github.com/claxima/HistologicalOntology/blob/master/CompetencyQuestions.pdf contains the complete document.

**Table 1:** Examples of CQs

Classification
What are the organs of the cardiovascular system?
What is the composition of the myocardium?
What are the muscular arteries?
Properties
What are the tunics in veins?
Which is the constitution of a media tunic?
What are the structures present in the large veins?
Constraints
A simple epithelial tissue cannot be stratified
A capillary is only composed of endothelium
An organ can have three tunics maximum
Inferences
If a set of cells is close to a light region, then the tissue is probably an
epithelial tissue
If an organ has a thin media tunic as well as a thick adventitia tunic and
a wide light region, it is probably a vein
If an organ has a thick media tunic and a small light region, it is probably
an artery

#### Classes and properties

In this step, we illustrate the construction of our ontology and explain its primitive classes and properties. The core classes of our histological ontology are: cells, tissues, organs and systems. These are the main structures to represent. Some examples of relevant properties are: layers, cell morphology, ducts, specialisation, mechanism of secretion, nature of secretion, valves and nodes. Some examples of object properties of histology ontology are included in Table 2.

**Table 2:** Object properties in histology ontology

Property	Domain class	Range class	Inverse property
isOrganOf	Organ	System	hasOrgan
isTypeOf	TypeOrgan	Organ	hasType
isCellOf	Cell	Tissue	hasCell
isMorphologyOf	Cell morphology	Epihelial tissue	hasMorphology
hasNumberLayer	Epithellial tissue	Number layer	isNumberLayerOf

A modular implementation taking into account tissues, organs and systems was used in our ontology to facilitate integration and/or reuse of histological data.

**Fig. 2:**
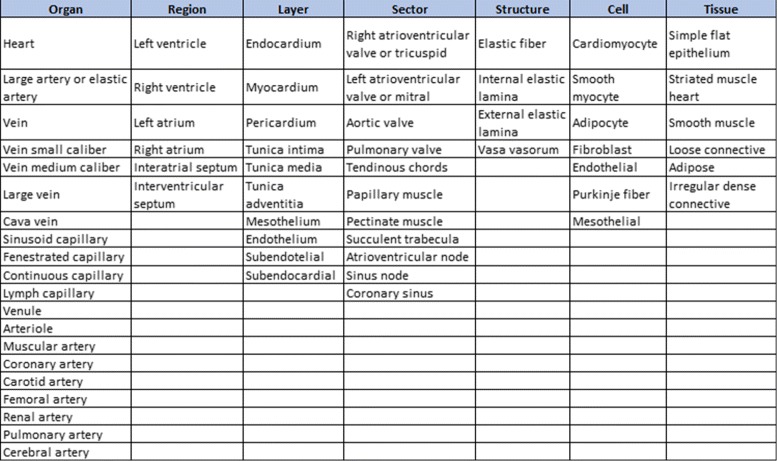
Glossary of human cardiovascular system

**Fig. 3:**
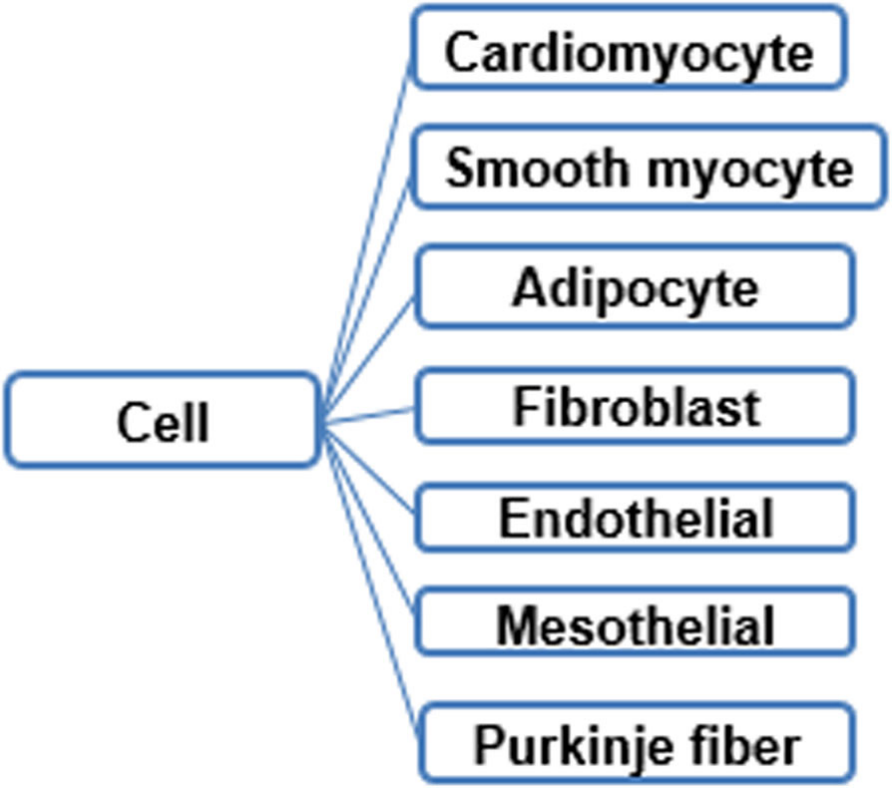
Taxonomy of main cells observed in a sample of the circulatory system

**Fig. 4:**
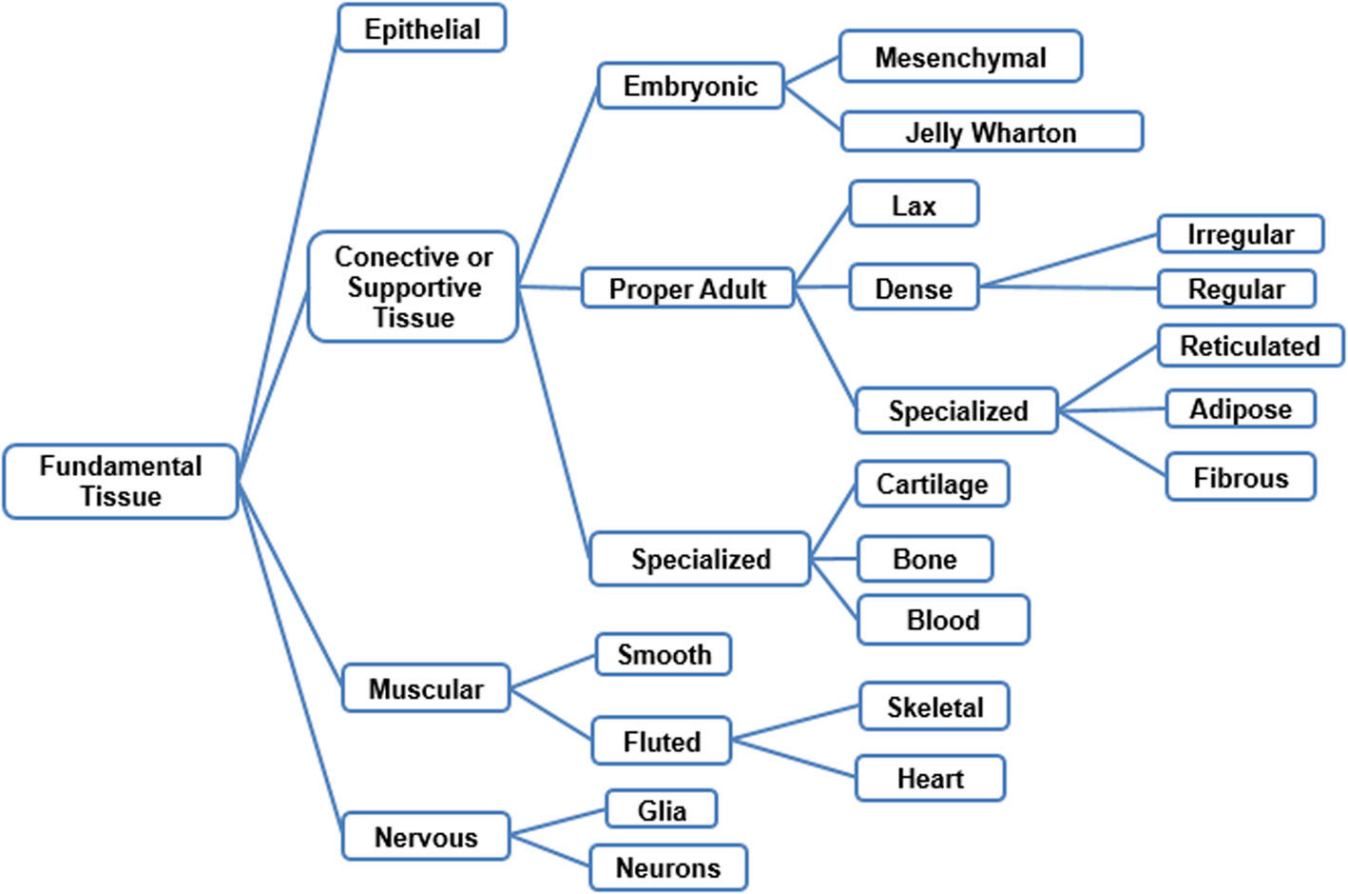
Taxonomy of the fundamental tissues. The epithelial tissue is not completely displayed here to improve visualisation

**Fig. 5:**
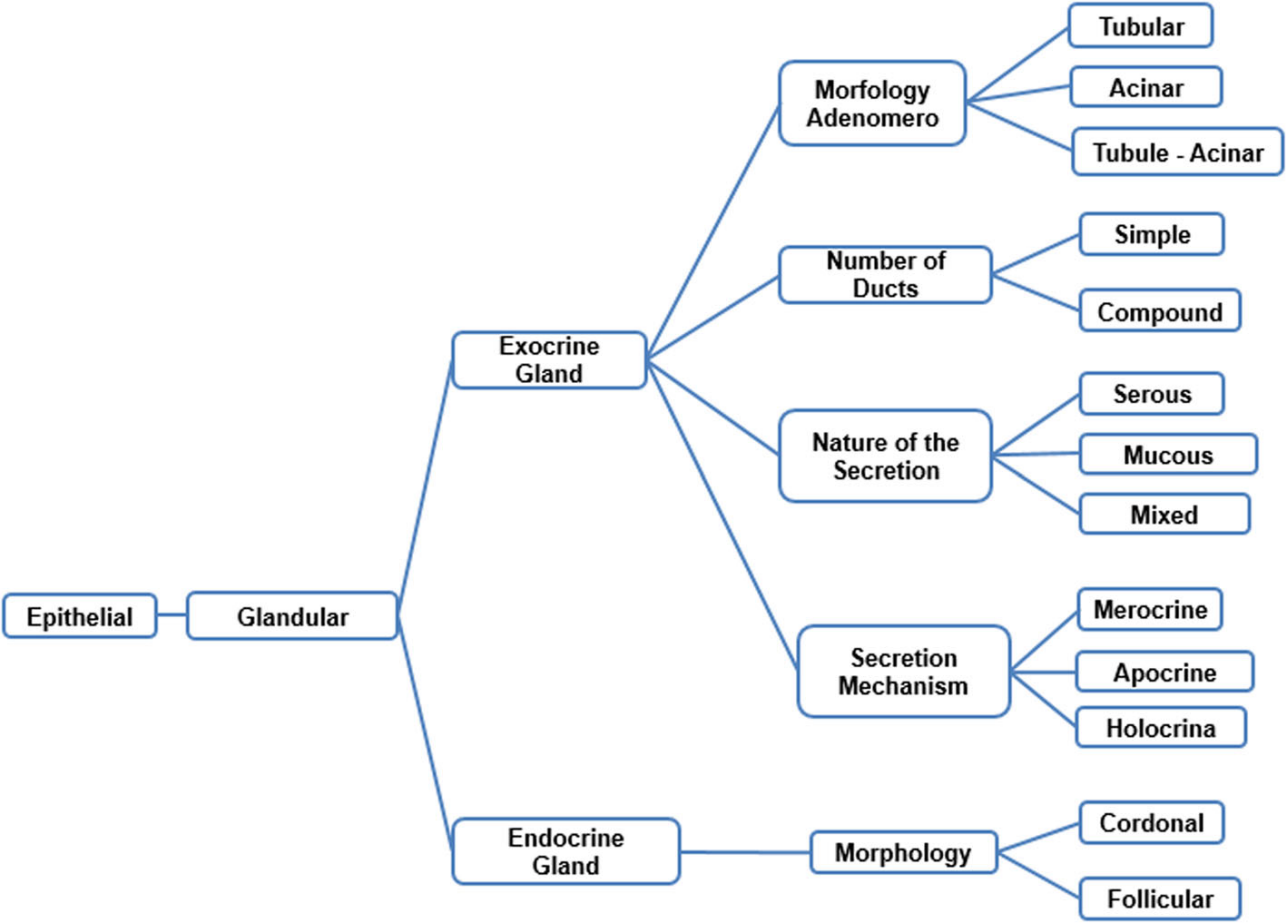
Taxonomy of the epithelial tissue

**Fig. 6:**
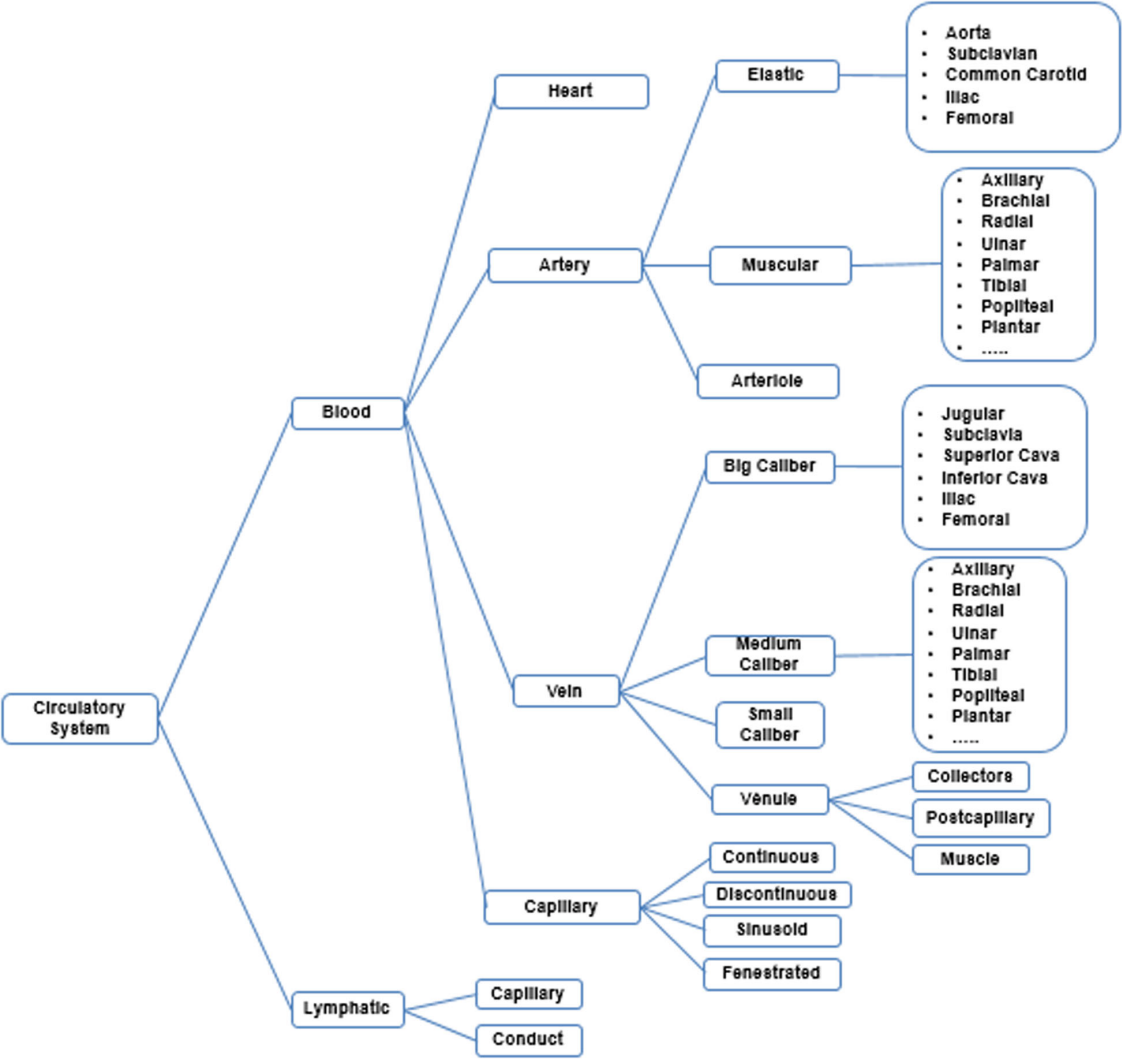
Taxonomy of histological classification of the circulatory system

**Fig. 7:**
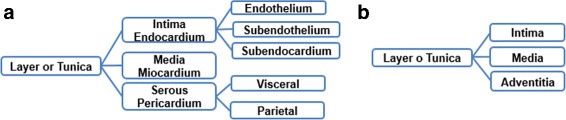
Taxonomy of histological classification of layers: **a** layers of the heart. **b** layers of blood vessels

**Fig. 8:**
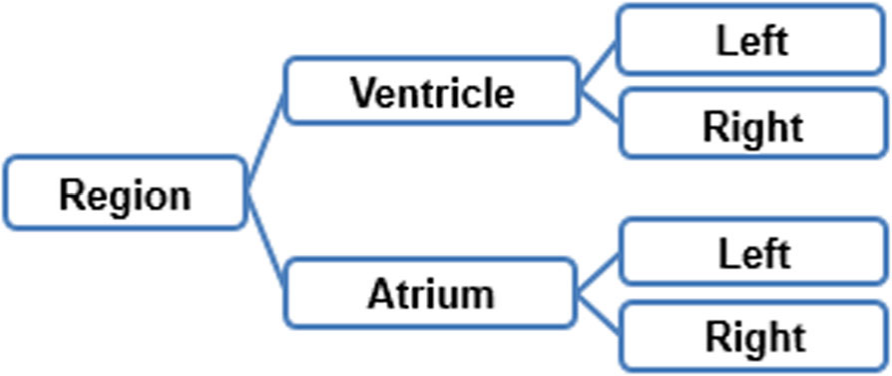
Taxonomy of classification of anatomical regions present in the heart

**Fig. 9:**
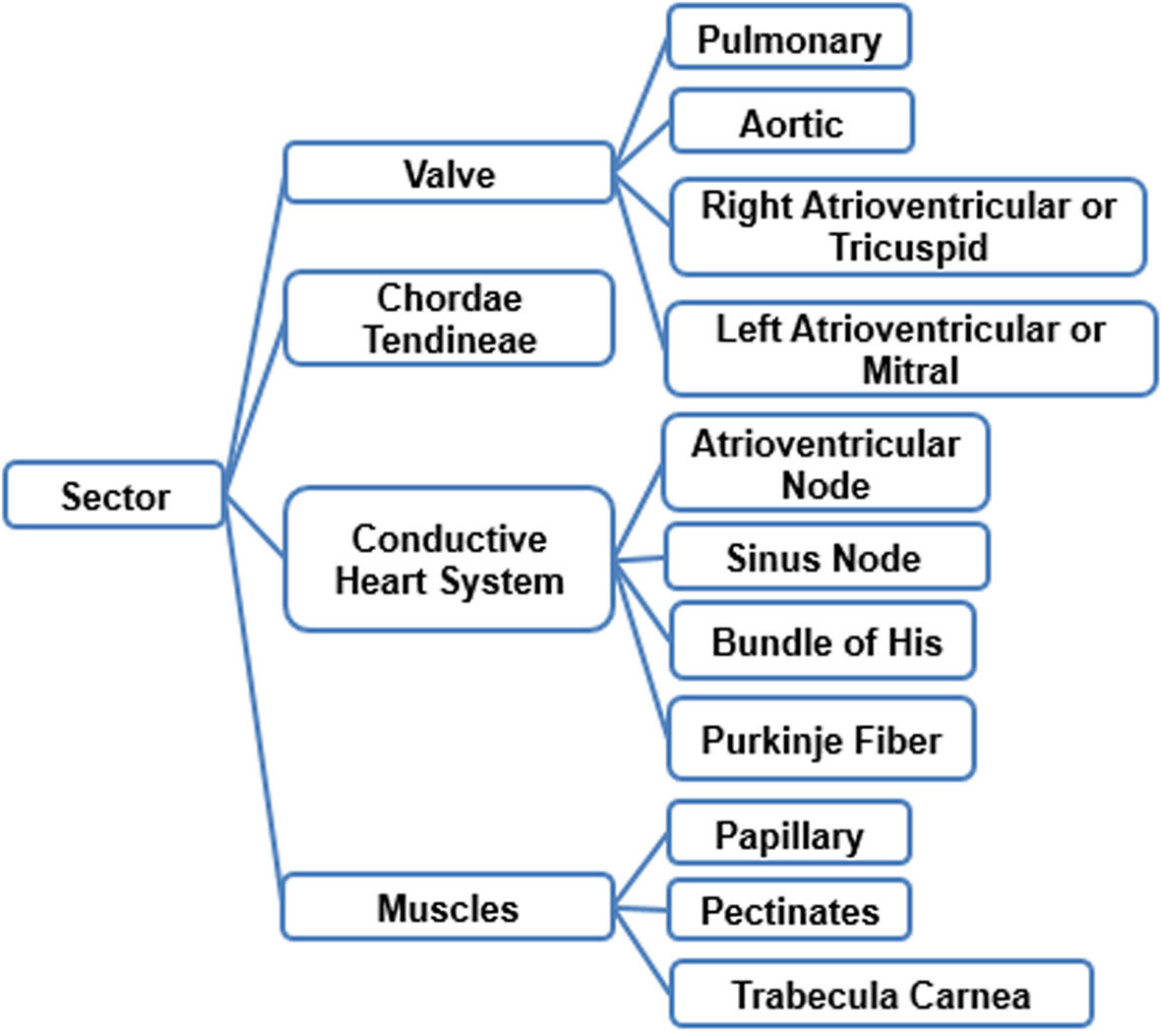
Taxonomy of classification of anatomical sectors present in the heart

Two tasks were developed in this stage: (i) build the glossary of terms with their definitions and synonyms, and (ii) build the taxonomy of concepts. Figure 2 shows the complete glossary of terms obtained for the human cardiovascular system. Figures 3, 4, 5, 6, 7, 8 and 9 show the CMs which represent the taxonomies for cells, tissues and organs; these taxonomies are divided to show in more detail the different components and relations.

### Identifying reusable ontologies

Ontology research and analysis were carried out to assess whether there were elements that could be reused in our proposal [22]. For that, we took into account the histological and the anatomical perspectives. BioPortal [23] contains some histological terms. However, this thesaurus has different kind of guidance to our research due to the fact that its organisation does not contain a specific order and some terms are randomly located, for this reason it cannot be reused. BioPortal [24] contains concepts similar to those required in our ontology such as tissues and cells. Nevertheless, this is a human histopathological ontology which contain abnormal cell types which can occur in either disease states or disease models, then this ontology cannot be used in our research. Additionally, this ontology does not contain the organs of the cardiovascular system nor the classification of tissues since it is focused on retinal, mammary, urethral, among others. Finally, some terms can be referenced as individual concepts. BioPortal [23] and [24] have similar terms to those required in our research, for instance terms related to the epithelial tissue. Nevertheless, these concepts are linked by a different route, tissues blood vessels. These ontologies contain many concepts but the hierarchical relations among them are not detailed in depth. Under this condition, if the hierarchy is represented as a tree, some of its branches are left inconclusive. This case is seen, for instance, for muscle tissue. Concepts are linked in one-way allowing to connect from a large to a small structure but not reverse. Due to the way the concepts are organised, the methods to search for a concept may not appear logical nor intuitive. Hence, the user may need specialised knowledge or spend more time and effort (e.g. exhaustive search) in finding possible routes for these terms. BioPortal [25] contains the cardiovascular system and its organs. It is a complete ontology and close to what is sought in our research. However, some terms are not in this ontology such as the type of epithelium, connective and muscle tissues, which has another classification — cutaneous, corneal and lymphatic. Moreover, it is a fairly complete cardiovascular system and organs ontology. It has large shortcomings regarding the fundamental tissues — epithelial tissue and muscle tissue can be referenced as individual terms. Uberon, the Uber-anatomy ontology, [17] is an anatomy ontology representing a variety of entities classified according to traditional anatomical criteria such as structure, function and developmental lineage. Uberon ontology takes into account Cardiovascular system. However, Uberon represents anatomical structures grouped in high-level categories and it is organised according to traditional anatomical classification criteria, being different to our histological classification criteria. BioPortal [26] is a mouse ontology with an adult gross anatomy focus, for this reason it does not contain microscopic terms such as cells, fibres, and tissue with histological information. However, this ontology contains some similar organ and system terms which can be referenced as individual concepts in our ontology.

Finally, we did not find in the State-of-the-Art an ontology of histology neither a similar organisation of hierarchies of histology terms that we may be able to reuse. We followed a ‘top-down’ approach [27] where histology experts work together to identify requirements and create the CMs. Finally, we did not reuse any available ontology, nevertheless, there is an open door to include terms which are related to existing ontologies by linking using *rdfs:sameAs* and *rdfs:seeAlso*.

### Iterative building of informal ontology models

**Fig. 10:**
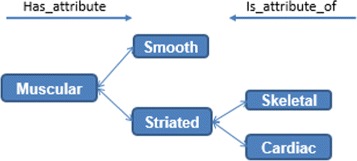
CM representation

We use CMs in each step of our methodology. CMs are graphs comprised of nodes connected by arcs representing concepts and relations between them [28] (see Fig. 10). CMs are useful to share and capture knowledge, to facilitate communication with experts as well as to formalise use cases, and for evaluation purposes. Figure 10 illustrates the classification of the muscular tissue, in two ways: (i) muscular tissue is classified into smooth and striated, (ii) striated muscular tissue is classified into skeletal and cardiac.

Histology and expert knowledge are represented using instances and relations with as much detail as possible in CMs. Concept-predicate structures are easily identified with this knowledge modelling. Subjects are entities that perform or receive an action, whereas the predicate is everything that may be said about a subject. The subjects, predicates and objects are extracted from histological knowledge manually.

Classes and subclasses were identified using the CMs representation; for example, epithelial tissue *is_a* fundamental tissue and simple flat epithelium *is_an* epithelial tissue. Similarly, attributes were obtained. For instance, *has_attribute or is_attribute_of*. An iterative process was carried out to represent histological and expert knowledge by providing a full narration of the instances, specific properties, and relations. Experts did a validation process after obtaining our representation of the knowledge.

### Formalisation

Informal models obtained, in the last step, with CMs are converted into formal models which are computationally valid, using Web Ontology Language Overview (OWL) [29]. Formal languages enable the encoding of knowledge and often include reasoning rules. Our histological ontology is expressed in OWL and implemented using Protégé [30].

The transformation from CMs models into an OWL model requires an interdisciplinary work. Domain experts develop part of the ontology by modelling their knowledge, with the assistance of knowledge engineers. Experts defined classes, properties and relations, with as much detail as possible, to obtain a consistent OWL model. Interdisciplinary work has advantages and challenges. One of the most important advantages is the possibility of covering topics in more depth, considering that there are many and varied perspectives for exploring a topic, to develop important discoveries. Challenges include arranging time for meetings, developing a common language and a knowledge baseline, dealing proactively with expectations and misunderstandings, focusing on a CM, and providing timely feedback.

## Results

In this section we present the results obtained using a three-fold approach to validate our ontology before putting it into use. First of all, we detected some of the most common pitfalls using *OOPS!*. Secondly, we performed expert evaluation using conceptual models. Thirdly, we evaluated how accurately the ontology answered our CQs.

### Detecting Pitfalls

We used *OOPS!* [31], a web tool for detecting the most common pitfalls in ontologies. *OOPS!* detects warnings in cases such as: reasoning problems, naming conventions, unconnected elements, modelling as well as reasoning problems and many others described in the catalogue. This evaluation enables to improve the maintainability, the accessibility and the clarity of the ontology.

**Fig. 11:**
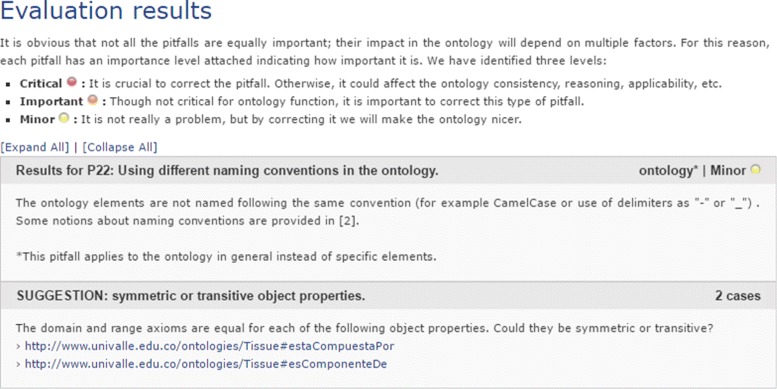
Evaluation results for tissues

**Fig. 12:**
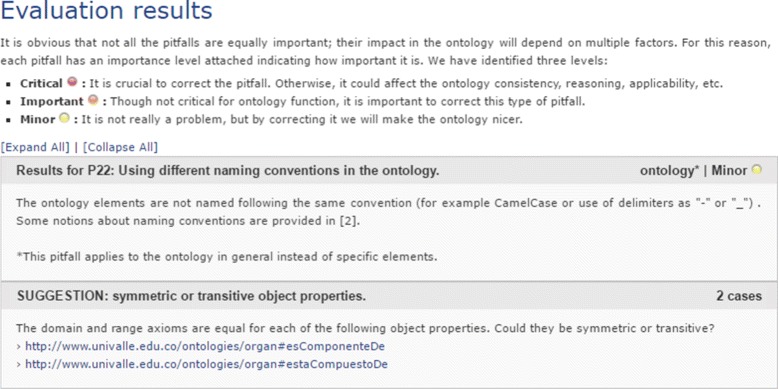
Evaluation results for organs and system

After executing *OOPS!* with the histological ontology, we obtained a summary of the pitfalls encountered as presenting in Figs. 11 and 12. Figures show two pitfalls being detected as well as one suggestion and one warning in each case.

### Expert evaluation

We use CMs for evaluating the ontology taking into account that CMs represent the conceptual scaffold of the knowledge we are representing. Although several criteria are used to validate ontologies, we are interested in the formal correctness of the ontology, as described in [32]: (i) completeness based on covering all terms related to the cardiovascular system, (ii) duplication errors to eliminate ontology elements which are redundant, (iii) disjunction errors to define a class as a conjunction of distinct classes, and (iv) consistency and coherence based on checking if the current definitions have been accurately represented — syntactically and semantically.

**Fig. 13:**
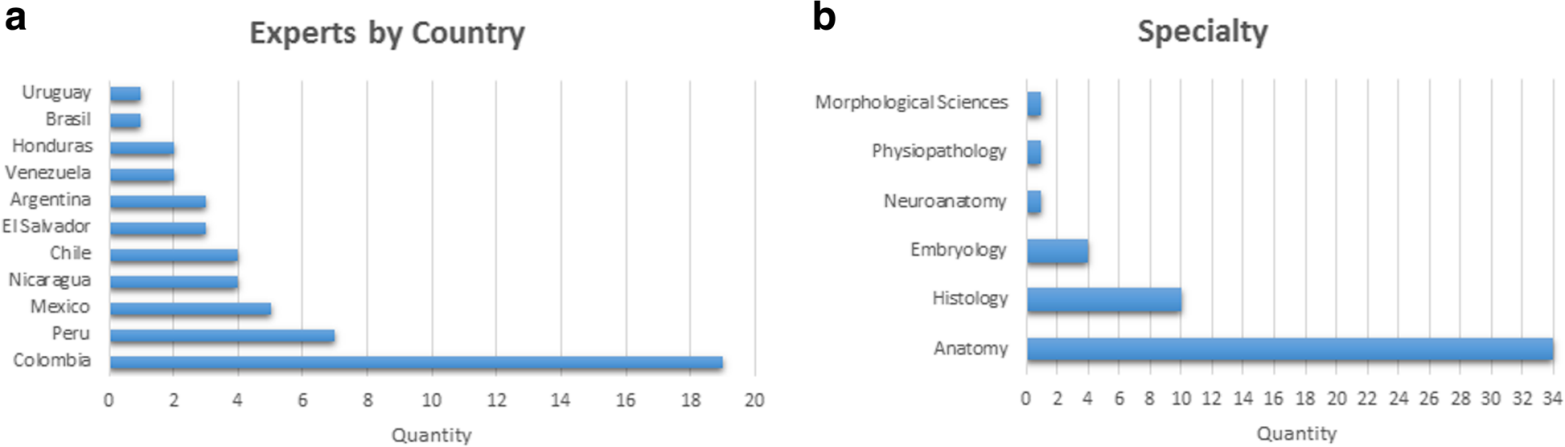
**a** Experts by country of the second survey. **b** Experts by specialty of the second survey. “Quantity” represents the number of experts

**Fig. 14:**
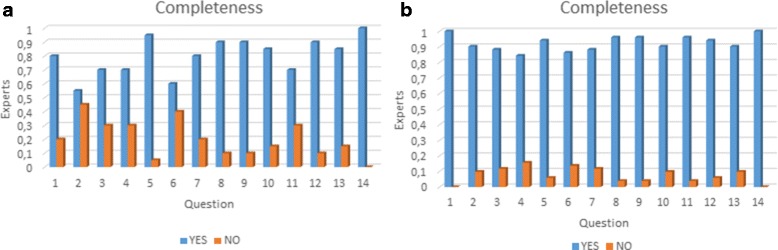
Completeness: **a** Results from the first survey. **b** Results from the second survey. In the axes: “Experts” represents percentage of experts per question and “Question” represents the associated number to a question

**Fig. 15:**
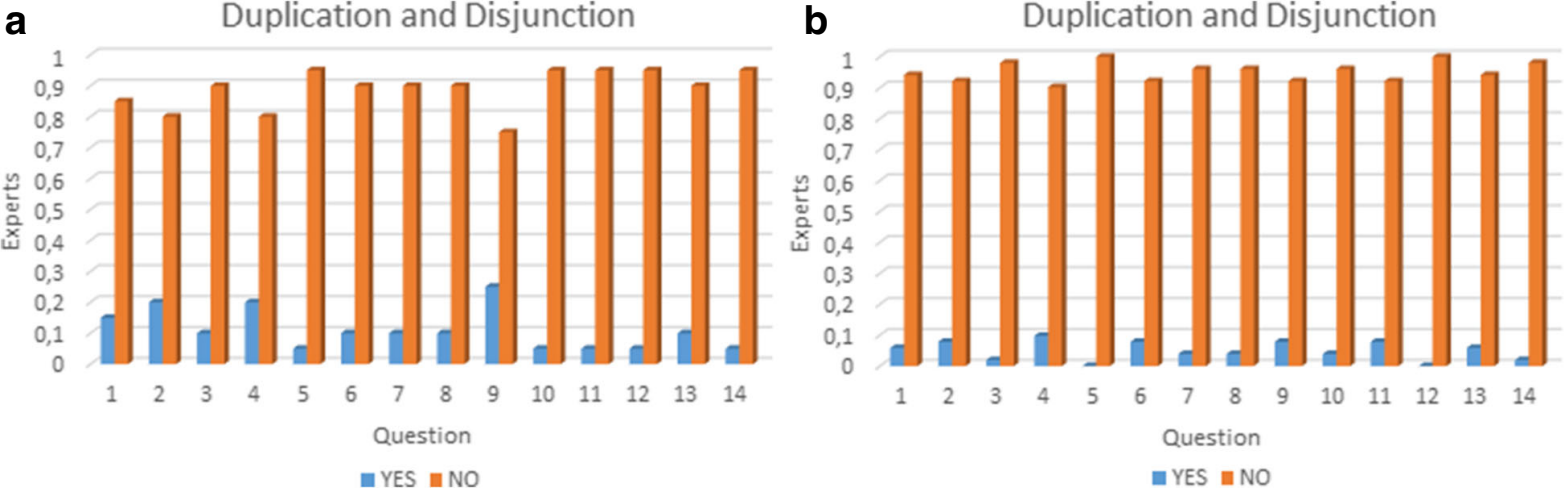
Duplication and disjunction: **a** Results from the first survey. **b** Results from the second survey. In the axes: “Experts” represents percentage of experts per question and “Question” represents the associated number to a question

**Fig. 16:**
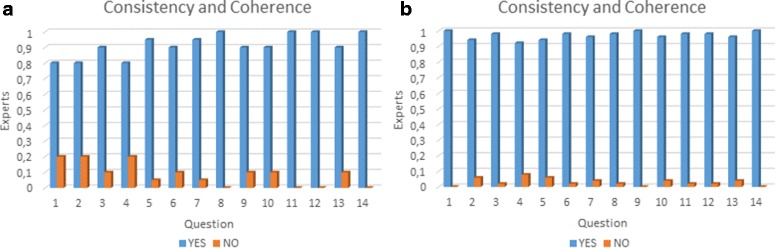
Consistency and coherence: **a** Results from the first survey. **b** Results from the second survey. In the axes: “Experts” represents percentage of experts per question and “Question” represents the associated number to a question

Abacha and Zweigenbaum [33] propose a validation of medical ontologies through simple questions with only two possible answers (Yes/No) and a textual feedback. This method makes the evaluation easier for medical experts and they can interpret feedback easier. We used this method through the construction of a survey. The elaboration of this survey was addressed with four basic objectives: (i) identify elements that need to be validated, (ii) organise the elements to be validated, (iii) identify the characteristics to be validated in these elements, and (iv)interpret the feedback and make the necessary updates. We have made the complete survey publicly available at the following URL http://survey-megaspace.rhcloud.com/survey/index.php/656146?lang=es. The second step consists in providing the survey to our group of experts. The third step consists in interpreting expert’s feedback to validate or modify the ontology. We applied two different surveys. The first survey was applied in order to do an initial evaluation on the first version of our ontology, which was enhanced following the expert recommendations. This survey was taken by 20 students in the third year of Medicine and Surgery at Universidad del Valle. The second survey was taken by 51 experts from Latin America with different specialties (See Fig. 13), from which 32 have over 10 years of experience. Additionally, the action fields are 22 professor, 1 researcher and 28 both. The results of the surveys are summarised in Figs. 14, 15 and 16. Taking into account our criteria to evaluate, the experts’ evaluation tackles issues concerning concepts and logical relations.

Where possible, the first version of the ontology was enhanced by following the student’s recommendations. However, one of the drawbacks of the first survey was the lack of experience of the participants. For this reason, their answers were previously revalidated by an expert in order to take them into account.

Each evaluated criterion increased, when it is compared to the first survey, by (i) completeness 35,196*%*, (ii) duplication and disjunction 17,156*%*, (iii) consistency and coherence 20,000*%*. The results confirm that the new version had improved regarding the first one using the experts’ suggestions. Additionally, our ontology was designed in a modular way that enables an easy integration or reuse. In this way, the integration of other systems, such as the digestive and the respiratory, can be done without modifying the cardiovascular system.

### Answering CQs

**Fig. 17:**
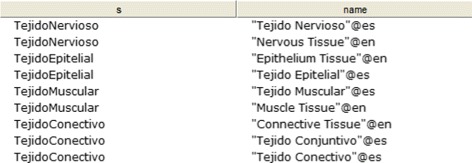
Obtained results for CQ-0



We evaluate the capability of the ontology to answer the CQs, using SPARQL [34]. SPARQL was used to represent the CQs to retrieve data from the ontology according to the query. SPARQL queries were created to verify if the ontology gives a correct answer for each CQ, https://github.com/claxima/HistologicalOntology/blob/master/SPARQL_Queries.pdf contains the complete document. CQ, SPARQL query and a figure with the result obtained are presented in the following examples:

CQ-0: What are the fundamental tissues? Figure 17 shows the obtained results.

**Fig. 18:**
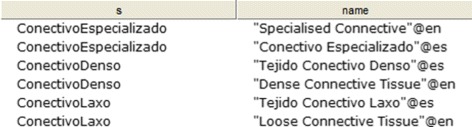
Obtained results for CQ-1



CQ-1: What are the types of connective proper tissue? Figure 18 shows the obtained results.

**Fig. 19:**
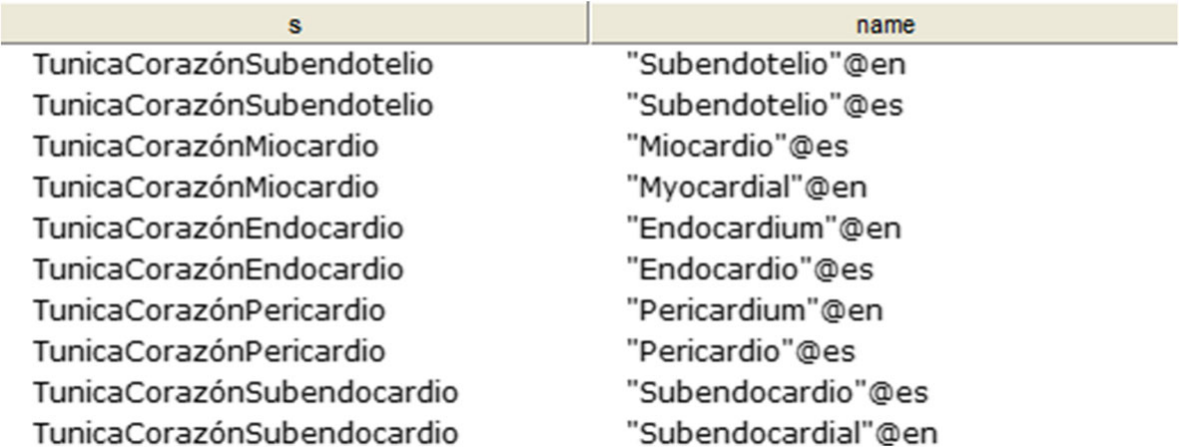
Obtained results for CQ-2



CQ-2: What are the layers present in the heart? Figure 19 shows the obtained results.

**Fig. 20:**
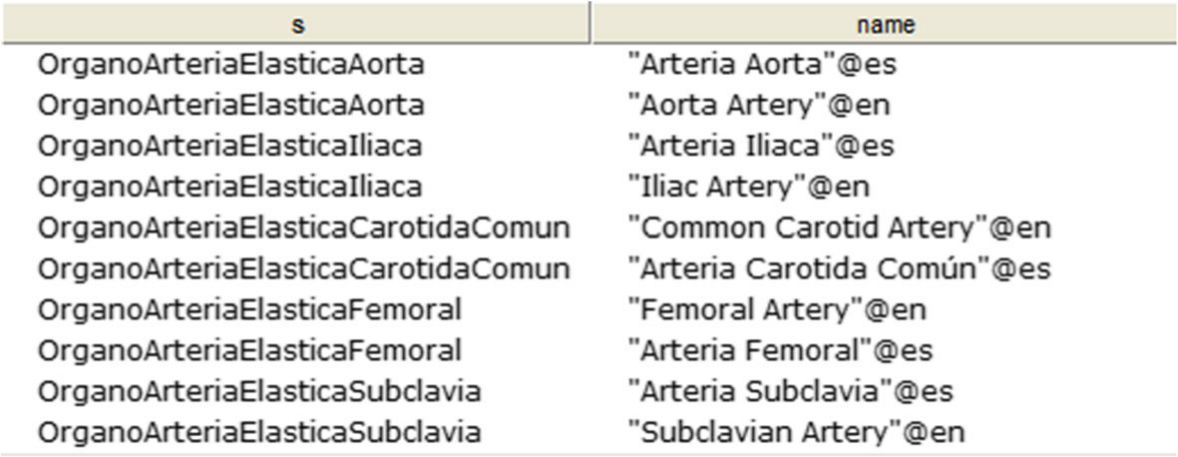
Obtained results for CQ-3



CQ-3: Which are the elastic arteries? Figure 20 shows the obtained results.

## Discussion

A three-fold approach to validate the histological ontology was used — detecting pitfalls using *OOPS!*, expert evaluation using CMs, and how accurately the ontology can answer the Competency Questions (CQ).

Regarding the detecting pitfalls, the results suggest that “the domain and range axioms are equal for two object properties” and a warning refers to the convention used. However, those are not pitfalls in our case and do not affect the correctness of our ontology. It does not represent a problem, since it is about appearance or style of the ontology and does not compromise the proper ontology functioning.

The results shown that the experts agreed with the following aspects of our ontology: completeness, duplication and disjunction, and consistency. Completeness was tackled by the first question in each CM; some relevant concepts were added to the ontology after the first evaluation. Duplication and disjunction were evaluated based on the second question at each CM and we have also ensured that there were neither duplication nor conflict in the concepts. Consistency and coherence were covered in the third question at each CM.

The obtained results in the experts survey were crucial for us due to the feedback provided based on the large experience in histology. This means that the feedback was valuable for our research and the fact that we obtained positive results makes it possible to put the ontology into use.

The criteria for an ontology evaluation (consistency, completeness, conciseness, expandability and sensitiveness) are used to addresses the possible types of errors made and the future use. Exist reliable indications of the quality of terms and definitions in ontologies and taxonomies [31]. However, the results obtained cannot be compared to other approaches in the state-of-the-art because these other works addressed different disciplines. Additionally, a key factor in the ontology evaluation is to evaluate and compare the ideas within the area [32].

## Conclusions

In this paper, we presented a histological ontology of the human cardiovascular system. The ontology enables to represent histological knowledge with the purpose of processing, inferring and obtaining new, and more complete, knowledge. The histological ontology was built from histological analysis perspective, potentiating its use in teaching, medical practices and biomedical research. We believe that our ontology meets the current need for teaching and learning the concepts of the cardiovascular system, using tissues without pathologies.

In the future, we will extend the ontology to other systems using the same methodology adopted for this ontology. Extending the ontology is possible taking into account that the ontology was implemented in a modular way — tissues, organs and systems. Moreover, we will use the ontology in four specific applications: (i) crossing-references to other ontologies in order to enable interoperability and integration among standards and applications, (ii) labelling and retrieval images of the BISCAR dataset [35, 36], (iii) refining the automatic classification of histological images of the human cardiovascular system [37] and (iv) teaching histology lectures at University of Valle using online histological images dataset (BISCAR) and the histological ontology. Additionally, since the development of a histological ontology was not our final goal, our future research will be focused on other applications of the ontology, such as supporting research in different ways.

## Endnotes

^1^ http://creativecommons.org/licenses/by/4.0/

^2^ https://sites.google.com/a/correounivalle.edu.co/grupo-de-tejidos-blandos-y-mineralizados/

^3^ www.univalle.edu.co

## Abbreviations

CMs: Conceptual models
CQs: Competency questions
*OOPS!*: Ontology Pitfall scanner!
OWL: Web ontology language overview
SPARQL: SPARQL protocol and RDF query language
